# A New Ligature

**Published:** 1904-02-27

**Authors:** 


### A NEW LIGATURE.

Mr. P. H. V. Hammerster writes: I shall be glad if you will allow me to bring before the notice of surgeons a ligature which has for some considerable time given me great satisfaction. It is made by a special process from numerous single cocoon fibres which produces a ligature equal in every respect to silk twist, and one which has the following advantages:—

Greater strength. Simplicity and ease of sterilisation—a few minutes' boiling renders it perfectly aseptic. Good keeping properties—it will keep indefinitely unimpaired in the wet or dry states. Absence of any tendency to slip when tied on a vessel or pedicle; owing to the flat form the ligature assumes it has no tendency to slip off. Ready temporary encapsulisation and perfect after absorption; the process of manufacture, whilst resulting in one thread of any desired thickness which retains its entirety, does not compress the component fibres together, so that the plastic lymph thrown out around the seat of application is able to penetrate between and around each strand composing the ligature; and to this is due, I believe, the absence of those annoyances so common with ordinary forms of ligature, namely, stitch abscess and the extrusion perhaps weeks and months after an operation of the deep ligatures.

The use of split-eyed needles with this as other forms of ligature saves both time and trouble.

The manufacturers have protected the ligature under their trade mark, and it can be obtained from Messrs. Maw, Son and Sons, who have provided a neat metal case containing varying sizes of the ligature, together with split-eyed needles adapted for each size, the whole being so constructed that, without disturbing the contents perfect sterilisation can be effected by a few minutes' boiling.

